# The diversity of PAH-degrading bacteria in a deep-sea water column above the Southwest Indian Ridge

**DOI:** 10.3389/fmicb.2015.00853

**Published:** 2015-08-25

**Authors:** Jun Yuan, Qiliang Lai, Fengqin Sun, Tianling Zheng, Zongze Shao

**Affiliations:** ^1^State Key Laboratory Breeding Base of Marine Genetic Resources, Key Laboratory of Marine Genetic Resources, Third Institute of Oceanography, State Oceanic Administration, Key Laboratory of Marine Genetic Resources of Fujian ProvinceXiamen, China; ^2^State Key Laboratory of Marine Environmental Science and Key Laboratory of MOE for Coast and Wetland Ecosystems, School of Life Sciences, Xiamen UniversityXiamen, China; ^3^Fujian Collaborative Innovation Center for Exploitation and Utilization of Marine Biological ResourcesXiamen, China

**Keywords:** Southwest Indian Ridge, deep-sea water column, PAHs, biodegradation, bacterial biodiversity, pelagic ocean

## Abstract

The bacteria involved in organic pollutant degradation in pelagic deep-sea environments are largely unknown. In this report, the diversity of polycyclic aromatic hydrocarbon (PAH)-degrading bacteria was analyzed in deep-sea water on the Southwest Indian Ridge (SWIR). After enrichment with a PAH mixture (phenanthrene, anthracene, fluoranthene, and pyrene), nine bacterial consortia were obtained from depths of 3946–4746 m. While the consortia degraded all four PAHs when supplied in a mixture, when PAHs were tested individually, only phenanthrene supported growth. Thus, degradation of the PAH mixture reflected a cometabolism of anthracene, fluoranthene, and pyrene with phenanthrene. Further, both culture-dependent and independent methods revealed many new bacteria involved in PAH degradation. Specifically, the alpha and gamma subclasses of *Proteobacteria* were confirmed as the major groups within the communities. Additionally, *Actinobacteria*, the CFB group and *Firmicutes* were detected. Denaturing Gradient Gel Electrophoresis (DGGE) analysis showed that bacteria closely affiliated with *Alcanivorax, Novosphingobium*, and *Rhodovulum* occurred most frequently in different PAH-degrading consortia. By using general heterotrophic media, 51 bacteria were isolated from the consortia and of these 34 grew with the PAH mixture as a sole carbon source. Of these, isolates most closely related to *Alterierythrobacter, Citricella, Erythrobacter, Idiomarina, Lutibacterium, Maricaulis, Marinobacter, Martelella, Pseudidiomarina, Rhodobacter, Roseovarius, Salipiger, Sphingopyxis*, and *Stappia* were found to be PAH degraders. To the best of our knowledge, this is the first time these bacteria have been identified in this context. In summary, this report revealed significant diversity among the PAH-degrading bacteria in the deep-sea water column. These bacteria may play a role in PAH removal in deep-sea environments.

## Introduction

Polycyclic aromatic hydrocarbons (PAHs) are a group of cytotoxic, mutagenic and carcinogenic compounds with two or more fused aromatic rings. They can be found in deep-sea sediments, such as those found 2.7 m beneath the bottom surface at a water depth of 3,962 m on the Middle Atlantic Ridge. This locations has a total PAH concentration (ΣPAHs) of 260 ∼440 ng/g dw (dry weight) (Cui et al., 2008; Wang et al., 2008). Recently, even higher PAH concentrations were detected in the hydrothermal vent area of the South Mid-Atlantic Ridge (Huang et al., 2014). There, the ΣPAHs ranged from 2768 to 9826 ng/g dry sediment at different sites (Huang et al., 2014). Among the PAHs detected in these reports, phenanthrene was most abundant. However, the origin and fate of PAHs in this extreme environment are not well understood.

Biodegradation of PAHs by bacteria has been investigated for a long time in coastal and gulf areas (Kanaly and Harayama, 2000; Harayama et al., 2004; Head et al., 2006; Yakimov et al., 2007; Kappell et al., 2014). Various PAH-degrading bacteria have been found in coastal environments (Head et al., 2006; Yakimov et al., 2007; Lamendella et al., 2014). Of these, bacteria of the genus *Cycloclasticus* are among the most wide-spread PAH degraders in marine systems (Dyksterhouse et al., 1995; Wang et al., 1996; Kasai et al., 2002; Yakimov et al., 2005; McKew et al., 2007; Teira et al., 2007). In deep-sea sediment, *Cycloclasticus* also can be found functioning as a PAH degrader (Cui et al., 2008; Wang et al., 2008).

In deep-sea water conditions, PAHs are thought to be absorbed on suspended particles and sink to the floor. As labile carbon sources in deep water are limited, PAHs in these environments could serve as valuable carbon and energy sources for bacterial growth. However, little is known about the diversity of PAH-degrading bacteria therein. In the present report, PAH degraders were examined at water depths ranging from 3946 to 4746 m on the Southwest Indian Ridge (SWIR). These bacteria displayed significant diversity and novelty, indicating the prevalence of PAH-degrading bacteria in the deep water system of the open sea.

## Materials and Methods

### Medium

For enrichments, a mineral salt medium (NH) was used that contained: 1.0 g NH_4_NO_3_, 0.5 g KH_2_PO_4_, 2.8 mg FeSO_4_, 1000 ml filtered sea water, adjusted to pH 7.5. PAHs were used as the sole carbon and energy source and included phenanthrene (>97%, Fluka), anthracene (>96%, Fluka), fluoranthene (≥98.5%, Fluka), pyrene (98%, Sigma).

Bacteria were isolated from enrichments by using two types of heterotrophic growth media, 216L and M2. The former medium contained: 1.0 g CH_3_COONa, 10.0 g Tryptone, 2.0 g Yeast extract, 0.5 g Sodium citrate, 0.2 g NH_4_NO_3_, 1000 ml sea water, adjusted to pH 7.5. The latter medium contained: 5.0 g CH_3_COONa, 0.5 g Tryptone, 0.5 g Yeast extract, 0.5 g Glucose, 0.5 g Sucrose, 0.05 g Sodium citrate, 0.05 g Malic acid, 1.0 g NH_4_NO_3_, 0.2 g NH_4_Cl, 0.5 g KH_2_PO_4_, 1000 ml sea water, adjusted to pH 7.6.

### Sampling and PAH Enrichment on Board

Water samples were collected from the site IR-CTD5 (31°0710′S, 58°9945′E) on the SWIR during the DY105-13 cruise of R/V “Da-Yang Yi-Hao” on December 7, 2005 (Supplementary Figure S1). Niskin bottles attached to a CTD (conductivity, temperature, and depth) circular rosette were used to collect water samples. The bottles were purchased from OceanTest Equipment, INC (Davie, FL, USA). The CTD instrument (SEB, Model: 13-02-B) was obtained from Seabird Electronics, INC (Bellevue, WA, USA). In each bottle, 20 L water was loaded at depths of 4746, 4696, 4546, 4396, 4296, 4196, 4146, 4096 and 3946 m. These depths are 20 to 820 m above the sea bottom, and the samples were numbered IR5-1 to IR5-9, respectively. The parameters recorded by the CTD at 3946 m and 4746 m were 0.9785°C, 11.2262 mg/liter DO, 3.47% salinity and 0.9316°C, 11.2415 mg/liter DO, 3.47% salinity, respectively.

To enrich the PAH-degrading bacteria, 400 ml water from each layer was drained into autoclaved bottles directly from the sampler. The enrichment process was initiated on board the boat at 20°C with PAHs as the carbon and energy sources. Samples were kept for 2 months until laboratory analysis.

### PAH Enrichment in the Laboratory

Polycyclic aromatic hydrocarbons dissolved in chloroform were added to flasks containing 150 ml autoclaved NH medium, with final concentrations of 100 mg/liter phenanthrene, 10 mg/liter of anthracene, 10 mg/liter fluoranthene, and 10 mg/liter pyrene. To evaporate the chloroform, flasks were shaken at 160 rpm for 2 days. Then, enrichment was initiated by adding 5.0 ml primary enriched cultures from each layer in the flasks, followed by shaking at 160 rpm in the dark at 25°C for 20 days. For a second round of enrichment in the lab, a 2.0 ml culture of each sample was transferred into the same medium in triplicate. In addition, each PAH was tested individually for degradation in parallel to the PAH mixture treatments. At days 4, 8, 12, and 20, 2 ml cultures of treatments with obvious bacterial growth were used for community genomic DNA extraction. At day 30, the residual PAHs in each flask quantified as described below.

### Bacterial Isolation from the PAH Enriched Consortia

After the samples were enriched by being transferred twice in lab, bacteria were isolated from PAH-degrading consortia by serial dilutions ranging from 10^-3^ to 10^-7^. These dilutions were then spread on both M2 agar and 216L agar plates and incubated at 18°C for ∼2 weeks. A total of 238 isolates were purified and then subjected to Rep-PCR analysis to eliminate redundant strains, as described previously (Ma et al., 2006). The isolates were then identified by 16S rRNA gene sequencing and analyzed using BLASTN at http://www.ncbi.nlm.nih.gov/BLAST. Nearly full length sequence of 16 rRNA gene was obtained by universal primer set 27f (forward) 5′-AGAGTTTGATCCTGGCTCAG-3′/and 1502r (reverse):5′ -ACGGCTACCTTGTTACGACT-3′. The ability of these bacteria to degrade PAH was tested with a mixture containing phenanthrene, anthracene, fluoranthene, and pyrene.

### Quantification of PAH Compounds

To assay the biodegradation capacity of the consortia, PAHs from the mixture were analyzed with Gas Chromatography Mass Spectrometry (GC-MS) after 1 month of incubation. The residual PAHs in each flask were extracted according to Guo et al. (2005). Simultaneously, 5 mg/liter fluorene was added as an internal standard of extraction. Then, degradation percentages were determined using GC-MS QP2010 (SHIMADZU) with a 30-m RTS-5MS capillary column (0.25 mm inside diameter and 0.25 μm film thickness). Details of thermal program and injection conditions were according to our previous report (Cui et al., 2008). Selected ion monitoring (SIM) mode was used for quantification of PAH, and full-scan mode mode was used for identification of PAH with the GC-MS Postrun Analysis software (Version 2.10, SHIMADZU).

### DNA Preparation

Total community DNA was collected from 2 mL culture of each sample on days 4, 8, 12, and 20 in sterile 2.0 ml Eppendorf tubes, and then immediately extracted. Genomic DNA of both consortia and isolates were extracted using the modified SDS-CTAB method (Sambrook et al., 1989).

### 16S rRNA PCR for DGGE Analysis

Polymerase chain reaction amplification of the V3 variable region of 16S rRNA fragments prior to Denaturing Gradient Gel Electrophoresis (DGGE) was performed as described by (Muyzer et al., 1993). To amplify the variable V3 region of bacterial 16S rDNA genes, the primer set was used: DGGEf (forward), 5′-CGCCCGCCGCGCGCGGCGGGCG GGGCGGGGGCACGGGGGGCCTACGGGAGGCAGCAG-3′, which contained a GC clamp linked to the 5′end; DGGEr (reverse), 5′-ATTACCGCGGCTGCTGG-3′.

All PCRs were performed with a Mastercycler (Eppendorf, Hamburg, Germany). Reaction mixtures contained 1.0 U of rTaq (Takara), each primer at a concentration of 0.5 μM, and 0.6 μl of template DNA in a total volume of 50 μl. After an initial 6 min denaturation at 95°C, a touchdown thermal profile protocol was used. The annealing temperature was decreased by 0.5°C per cycle from 65 to 55°C, followed by 20 additional cycles at 55°C. Amplification was carried out with 1 min of denaturation at 95°C, 1 min of primer annealing, and 1.0 min of primer extension at 72°C, followed by a final extension at 72°C for 10 min. PCRs were carried out in replicates of four using the consortium DNA, then the mixtures were combined PCR amplifications were checked on 1% (w/v) agarose gel prior to DGGE analysis.

### Denaturing Gradient Gel Electrophoresis

The purified PCR product was loaded onto 8% (w/v) polyacrylamide gels (ratio of acrylamide to bisacrylamide, 37.5:1). The denaturing gradients used ranged from 30 to 70% denaturant [100% denaturant contained 7 M urea and 40% (v/v) formamide]. The gels were electrophoresed using a D-Code instrument (Bio-Rad) in 1 × TAE buffer (40 mM Tris-acetate, 1 mM Na-EDTA, pH 8.0) running at 30 V for 15 min first and then 130 V for 4.5 h at 60°C.

After electrophoresis, the gel was stained with ethidium bromide (10 μg/ml) for 15 min, and images were captured using an Alpha-Imager Imaging System with AlphaEase FC image software 4.1.0 (Alpha Innotech).

Each visible band on the DGGE gels containing the PAH-degrading consortia was excised manually, and the DNA was extracted using the method described by Muyzer et al. (1993). Using 5 μl of the extracted DNA as template, PCR was carried out to amplify target DNA for cloning. Positive PCR products were purified using the E.Z.N.A Cycle-Pure Kit (OMEGA Bio-tek, USA) and cloned into the pMD19-T Vector (Takara). After being confirmed using another DGGE gel, they were sequenced with an ABI model 3730 DNA sequencer (Invitrogen Shanghai).

### Nucleotide Sequence Deposit

The 16S rRNA sequences of bacteria isolated from the nine consortia have been deposited in the GenBank database (http://www.ncbi.nlm.nih.gov/BLAST) under accession No. EU440952∼EU441002. The DGGE band sequences determined in this study have also been submitted to the GenBank database and have been assigned accession numbers EU441003∼EU441036.

## Results

### PAH Degradation by Deep Water Layers

**FIGURE 1:**
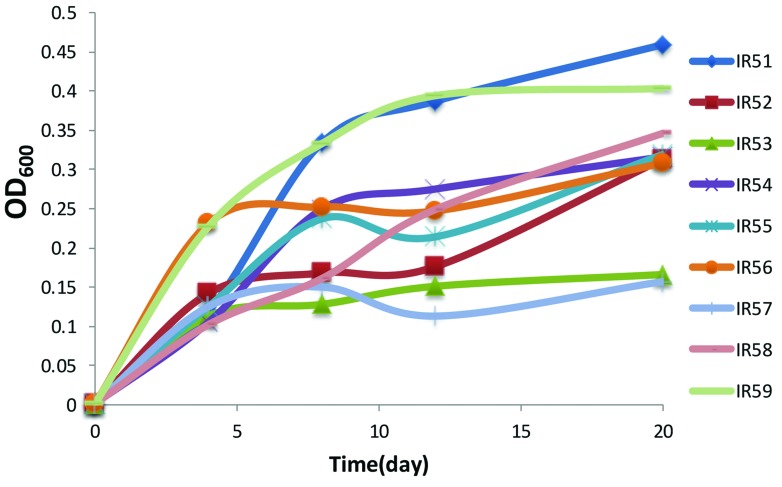
**The growth of the nine consortia on a polycyclic aromatic hydrocarbon (PAH) mixture of phenanthrene (100 mg/liter), fluoranthene (10 mg/liter), anthracene (10 mg/liter), and pyrene (10 mg/liter).** Cell growth was determined by OD_600_ from 4 to 20 days. Incubation was at 28°C in the dark in a constant shaker.

After the initial 2 months enrichment with a PAH mixture of phenanthrene, anthracene, fluoranthene, and pyrene as the sole carbon and energy sources, PAH degradation occurred in all treatment conditions of nine layers of water samples acquired from 3946 to 4746 m. Obvious bacterial growth was observed in all the enrichment cultures with the color changing to dark brown. In the lab, the initial enrichment cultures were transferred to fresh NH medium with PAHs as the sole carbon source (with 5% inoculum). The resulting final nine consortia were numbered IR51–IR59 (IR5, representing the sampling site IR-CTD-5; 1-9, the layer number based on the water depths listed above). The growth curves of all cultures during the second round of enrichment in the lab are shown in **Figure 1**. Of these cultures, layer 1 (IR51, 4746 m) showed the best growth with the highest cell concentration (OD_600_ = 0.459) at day 20, followed by layer 9 (IR59, 3946 m) (OD_600_ = 0.400) (**Figure 1**).

**FIGURE 2:**
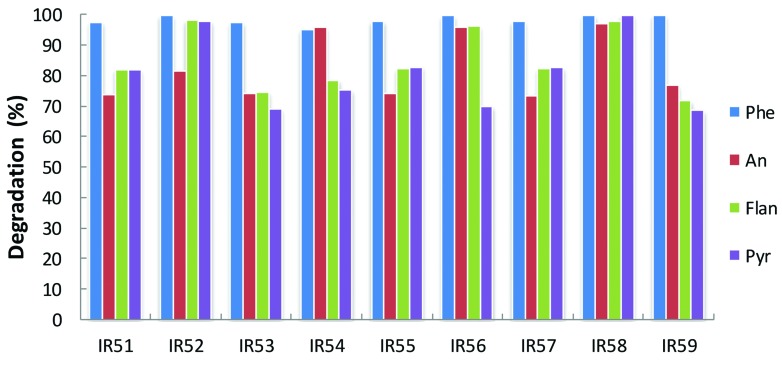
**Degradation of the 4-PAH mixture by nine consortia from the deep sea water column of the Indian Ocean.** PAH degradation experiments were conducted in 100 ml Erlenmeyer flasks containing 30 ml NH medium with a mixture of 4-PAHs as carbon sources (using the same concentrations in **Figure 1**). The residual PAHs were quantified with GC-MS; the degradation percentage was the average of triplicate repeats at 30 days. Phe, An, Flan, and Pyr are abbreviations of phenanthrene, anthracene, fluoranthene, and pyrene, respectively.

Meanwhile, PAH degradation was further confirmed by GC-MS quantification. As shown in **Figure 2**, phenanthrene was almost completely degraded in all cases after 30 days incubation, while anthracene, fluoranthene, and pyrene showed degradation ranging from 69 to 99%.

### Isolation of PAH-Degrading Bacteria

**FIGURE 3:**
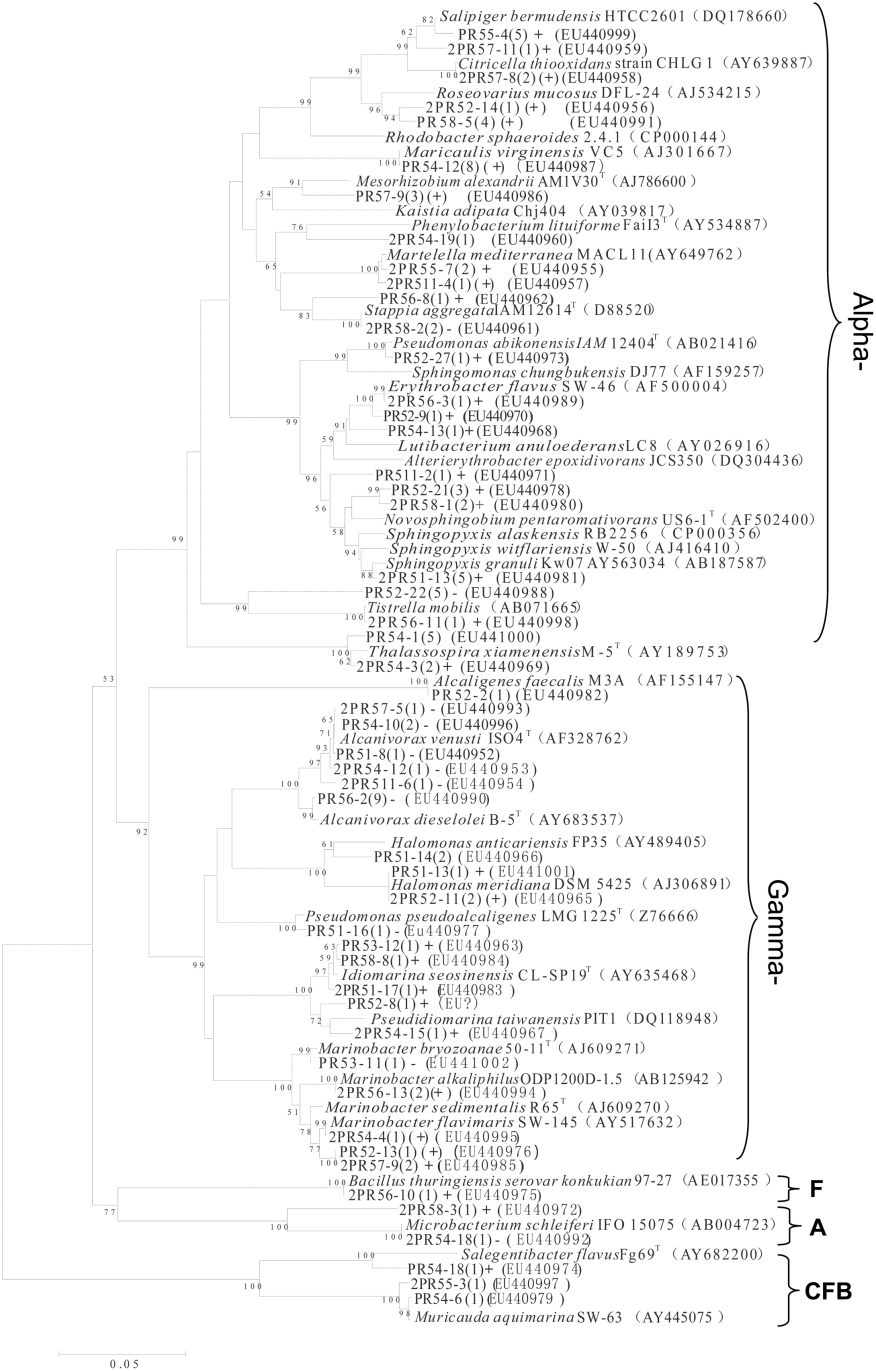
**Phylogenetic tree of culturable strains isolated from the nine PAH-degrading bacterial consortia.** The dendrogram was constructed from a matrix by the neighbor-joining method using the MEGA3.1 program. Thousands of trial bootstrap analysis was used to provide confident estimates for phylogenetic tree topologies. The scale bar represented 0.05 substitutions per nucleotide site. The number in the bracket shows the frequency with which the bacterium appeared in the nine PAH-degrading consortia. “+” means the bacterium can grow well in the medium with PAHs as carbon sources. “(+)” means the bacterium can grow but not very well in the medium with PAHs as carbon sources. “-” means the bacterium cannot grow in the medium with PAHs as the carbon sources. Subgroups: Alpha-, *Gammaproteobacteria*; F, Firmicutes; A, Actinobacteria. Isolates named as PR and 2PR were isolated on agar plates of M2 agar and 216L, respectively.

From the nine consortia, 238 isolates were purified. Of these, 168 strains showed a unique Rep-PCR pattern (not shown), 51 of which varied in their 16S rRNA gene sequences (**Table 1**). Phylogenetic analysis showed that they belonged to 29 genera. As shown in the phylogenetic tree, most belonged to *Proteobacteria* (88% of the total), in addition to *Actinobacteria* (4.0%), the CFB (Cytophaga-Flexibacter-Bacteroides) group (6%), and *Firmicutes* (2%). The largest subgroup was the *Alphaproteobacteria*, containing 18 genera of *Erythrobacter, Thalassospira, Martelella*, *Novosphingobium, Pseudaminobacter, Tistrella, Salipiger, Sphingomonas, Sphingopyxis*, and *Stappia*. The *Gammaproteobacteria* we detected contained *Alcaligenes*, *Alcanivorax*, *Halomonas*, *Idiomarina*, *Marinobacter*, *Pseudidiomarina*, and *Pseudomonas*. In addition, isolates closely related to *Muricauda* and *Salegentibacte* in CFB group, a bacterium belonging to the genus *Microbacterium* in the *Actinobacteria* group and an isolate of *Bacillus* were also found. Among these, 13 appear to be novel species, with similarities to the 16S rRNA sequences of known species ranging from 88.6 to 97.3%. In GenBank, the closest relatives of these 13 species belonged to *Alterierythrobacter, Halomonas, Kaistia, Pseudaminobacter, Phenylobacterium, Pseudidiomarina, Roseovarius, Rhodobacter, Salegentibacte, Sphingomonas, Sphingopyxis*, and *Stappia* (**Figure 3**).

**Table 1:** The bacteria detected by Denaturing Gradient Gel Electrophoresis (DGGE) in PAHs- and Phe-degrading consortia.

Closest relatives in GenBank^∗^	Accession No.	Similarity (%)	Corresponding bands^#^		Corresponding isolates
			IR51-59	IR51P-59P	
*Alcanivorax dieselolei* B-5	AY683537	99.47	**1-2**, **2-3**, **3-3**, **4-4**, **5-5**, **7-3**, **9-3**	**P1-3**, **P2-4, P4-1**, **P5-2,** P6-3, P7-1, **P8-3**, **P9-1**	/
*A. dieselolei* B-5	AY683537	96.87	/	**P2-2**	/
*A. dieselolei* B-5	AY683537	98.43	/	P2-5, P6-4	/
*Alcanivorax* sp. EPR 7	AY394866	100.00	3-4, **8-2**	**P8-4**, **P9-2**	/
*A. venusti* ISO4	AF328762	98.09	9-5	P5-5, P9-3	2PR511-6
*A. venusti* ISO4	AF328762	100.00	**5-9**	/	PR51-8
*Arthrobacter* sp. 255-8a	AY444852	90.68	**3-7**, **4-8**	P4-5	/
*Arthrobacter* sp. 255-8a	AY444852	91.27	3-6, **4-7**	P4-4	/
*Arthrobacter* sp. 255-8a	AY444852	96.46	**8-4**	P8-5, P9-4	/
*Arthrobacter* sp. ADG1	AY651318	91.27	**2-5,** 6-7	P1-5, P2-8, P5-6, **P6-7**	/
*Azospirillum* sp. 5C	AF413109	95.21	**1-8**, **3-10**, 4-10, 5-14	/	/
*Bacillus subtilis* B-2009	AM110937	99.40	2-8, 3-9, 7-8	P3-9, P7-10	/
*Bartonella* sp. AD273	DQ113447	98.79	1-4, **6-8**, **7-6**, 8-5	P6-8, P7-8, P8-6, P9-5	/
*Citricella thiooxidans* CHLG 1	AY639887	98.81	**2-7**, 3-8	P1-6, P3-7, P4-6	/
*C. spirillensus* M4-6	AY026915	100.00	**2-1**, 3-1, **5-2**	P1-1, P2-1	/
*Erythrobacter* sp.G265	AY371411	99.26	/	**P3-3**	/
*Erythrobacter* sp.G265	AY371411	100.00	6-4	**P6-2**	2PR56-3
*Halomonas aquamarina* DSM 30161	AJ306888	100.00	**9-6**	/	PR51-13
*Halomonas meridiana* DSM 5425	AJ306891	99.88	8-6	/	2PR52-11
*Halomonas* sp. 3029	AM110984	100.00	7-7	P7-9, P8-7	2PR52-11
*Maricaulis virginensis* VC5	AJ301667	100.00	8-7	/	PR54-12
*Marinobacter aquaeolei* OC-11	AY669171	99.19	2-2	P2-3, P3-2	/
*Microbacterium schleiferi* DSM 20489	Y17237	100.00	**4-9**	/	2PR54-18
*Novosphingobium aromaticivorans* DSM 12444	CP000248	98.79	**5-8**, 6-5, **8-3**	**P3-5**	/
*N. aromaticivorans* DSM 12444	CP000248	100.00	**9-4**	**P5-4**, **P6-5**, **P7-5**	2PR58-1
*N. aromaticivorans*5-8B	DQ768707	100.00	4-2	/	/
*Novosphingobium hassiacum* W-51	AJ416411	95.81	**2-4**	P1-4, P2-6	/
*Novosphingobium pentaromativorans* US6-1	AF502400	98.64	**1-3**	/	/
*Novosphingobium* sp. MG36	AJ746093	98.77	3-5, **4-6**, **6-6**	**P2-7**, **P4-3**	/
*Novosphingobium* sp. MG36	AJ746093	100.00	/	**P6-6**, **P7-6**	PR52-21
*Porphyrobacter* sp. J3-AN66	DQ454121	98.79	7-1	/	/
*Pseudaminobacter salicylatoxidans* BN12	AF072542	95.60	**1-5**, 2-9, 3-11, 5-11	**P1-7**, P3-10	/
*Pseudomonas* sp. BJC3	DQ834358	100.00	1-1	P1-2	/
Rhizobiales bacterium RR47	AB174816	93.08	**4-5**, 5-7	P3-4, **P4-2**, P5-3, P7-4	/
*Rhizobium undicola* Liujia-81	DQ648579	92.90	4-1, 5-1, 6-1, 8-1, 9-1	**P8-1**	/
Rhodospirillaceae bacterium PH30	AF513476	99.30	/	P3-8	/
*Rhodovulum* sp. AR2002	AJ401214	95.21	1-7, **2-11**, 3-12, 5-13	P1-8, P3-11	/
*Rhodovulum* sp. AR2002	AJ401214	96.75	**1-6**, **2-10**, 5-12, 6-10, 9-7	P5-8, P6-10, P9-6	/
*Rhodovulum* sp. AR2002	AJ401214	95.81	5-10	P5-7	/
*Shewanella oneidensis* 84F	AB182078	91.09	3-2, **4-3**, 5-3, 6-3, 7-2	P5-1, P6-1	/
*Sinorhizobium medicae* M58	DQ423248	100.00	6-9	P6-9	PR57-9
*Novosphingobium* sp. FND-3	DQ831000	99.40	5-4	/	/
*Sphingomonas* sp. PXM	AY232825	98.41	7-4	P7-3	/
*Sphingomonas* sp. SAFR-042	AY167827	99.30	/	**P3-6**	/
*Sphingomonas* sp.MD-1	AB110635	98.82	6-2	**P7-2**	/
*Thalassospira lucentensis* DSM 14000	AM294944	99.39	**7-5**	P7-7	PR54-1, 2PR54-3
*Zymomonas mobilis* ATCC29191	AF281034	97.00	**2-6**	/	/

*^∗^Reference bacteria listed in order of alphabet. ^#^The boldface bands in blue were the predominant member in their own consortium.*

In addition to phylogenic analysis, these 51 isolates were also tested for PAH-degradation. We found that 34 isolates could grow with PAHs as the sole carbon and energy sources (labeled with “+” in **Figure 3**). Among them, 20 bacteria belonged to *Alphaproteobacteria*. These 34 degraders contained some bacteria that have been previously reported, such as *Bacillus* (Hunter et al., 2005), *Novosphingobium* (Sohn et al., 2004), *Lutibacterium* (Chung and King, 2001), *Sphingomonas* (Bastiaens et al., 2000), and *Marinobacter* (Hedlund et al., 2001). However, many others had not been reported to be PAH degraders prior to this study. This latter group included *Alterierythrobacter, Citricella, Erythrobacter, Idiomarina, Maricaulis, Martelella, Pseudidiomarina, Rhodobacter, Roseovarius, Salipiger, Sphingopyxis*, and *Stappia.*

### Bacterial Community Composition of the PAH-Degrading Consortia

**FIGURE 4:**
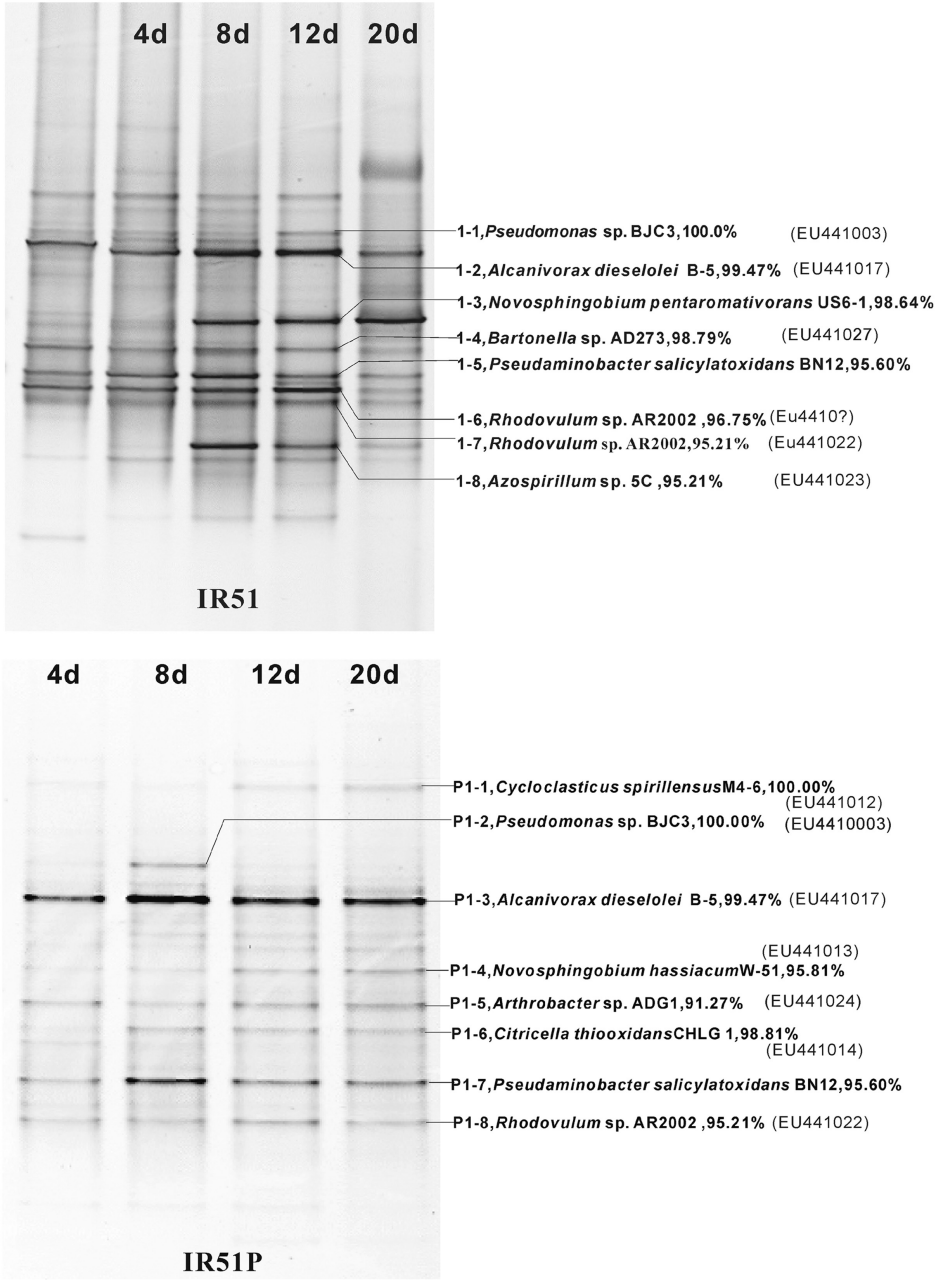
**Community structures of the PAH-degrading consortia from 4746 m water depth (20 m to the sea floor).** Numbers (4–20 days) above each lane indicate the number of days inoculated. The first lane is the initial inoculum of the last transfer. The closest described relatives in GenBank of the main bands in profiles are labeled at the right. **(Top)**, IR51grown with 4-PAH mixture; **(Bottom)**, IR51P grown with phenanthrene.

The bacterial composition of all consortia changed from the first day to the 20th day of the incubation process and also varied from layer to layer (**Figure 4**, Supplementary Figures S2 and S3). For an example, the consortium of IR51 that was generated from the first layer contained at least seven major bands (Band 2 to Band 8), as shown in the DGGE profile in **Figure 4**. These bands belonged to the genera *Alcanivorax, Novosphingobium, Bartonella, Pseudaminobacter, Rhodovulum*, and *Azospirillum.* Two bacteria, *Novosphingobium pentaromativorans* US6-1^T^ (98.64%; Band1-3, with “1-” indicating the first layer) and *Azospirillum* sp. 5C (95.21%; Band1-8), became predominant at day 8. The bacterium of Band1-3 remained a predominant member throughout the time course, while the abundance of Band1-8 decreased after day 8. At day 20, *Novosphingobium* (Band1-3) turned out to be the most predominant member, indicating a key role in PAH degradation. In addition, Band1–2, closely related to *Alcanivorax dieselolei* B-5 (99.47%), was also an important member and was a major band from days 8 to 12 (**Figure 4**). This bacterium reoccurred as the most dominant member in the community with phenanthrene as the sole carbon and energy source (**Figure 4**); however, it failed to degrade any PAHs by itself.

The community structures of the other water layers (IR52–IR59) were also examined (Supplementary Figures S2 and S3). Consistent with the results above, *Alcanivorax* and *Novosphingobium* were the most common bacteria in all the consortia. For an instance, an isolate closely related to *A. dieselolei* B-5 was a predominant member of almost all consortia. Bacteria of the genus *Novosphingobium* were also primary members of consortia from all depths and were closely related to *Novosphingobium aromaticivorans, N. hassiacum, N. pentaromativorans*, and *Novosphingobium* sp. MG36.

Interestingly, some novel species were detected as key members of the PAH-degrading consortia. For example, those with only 90.7–96.5% similarity to *Arthrobacter* sp. 255-8a (AY444852), as well as the bacteria with only 95.2–96.7% similarity to *Rhodovulum* sp. AR2002 and the bacterium with only 93.1% similarity to *Rhizobiales* bacterium RR47, occurred as a strong band in consortia IR53, 54, and 58. These bacteria have never been reported to be PAH degraders. Conversely, *Cycloclasticus spirillensus* (100.00%), the most famous PAH-degrading bacterium in marine environments (Head et al., 2006), occurred in consortia IR52, IR53, and IR55 (Supplementary Figures S2 and S3), but was not as important here as *Novosphingobium*.

### Single PAH Degradation

To detect the degradation of a single PAH, phenanthrene, pyrene, anthracene, and fluoranthene were used separately as the sole carbon source. Unexpectedly, none of the nine consortia could grow with anthracene, fluoranthene, or pyrene. However, all consortia could grow with phenanthrene.

During the degradation of phenanthrene, the community structure was relatively stable and not as dynamic as communities with the PAH mixture. Additionally, the bacterial composition was notably simplified with phenanthrene alone. The DGGE profile of consortium IR51P is shown in **Figure 4**, and other consortia are displayed in Supplementary Figure S3 (IR52P–IR59P). Consistent with results in IR51 (**Figure 4**), the predominant band in the other consortia was *A*. *dieselolei* B-5 (99.47%; Band P1-3). However, another predominant member, *N. pentaromativorans* US6-1^T^ (98.64%; IR51, Band1-3, **Figure 4**), was replaced with *N. hassiacum* (95.81%; IR51P, Band P1-4, **Figure 4**). In addition, *C. spirillensus* (100%; Band P1-1) was present. The bacterium of *Pseudomonas* sp. BJC3 (100.0%; Band P1-2) occurred with both phenanthrene alone and the consortia grown with the PAH mixture.

Some bacteria occurred only in the phenanthrene consortia, such as *Erythrobacter* sp.G265 (99.26%; Band P3-3), *Sphingomonas* sp. SAFR-042 (100%; P3-6) and *Novosphingobium* sp. MG36 (100%; P6-6) (**Table 1**). The most prevalent bacteria involved in phenanthrene degradation also belonged to *Novosphingobium* and *Alcanivorax*.

## Discussion

Oil spilling into the Gulf of Mexico from the Deepwater Horizon blowout stimulated indigenous bacteria of the order Oceanospirillales in the *Gammaproteobacteria*, which are likely involved in alkane degradation in the deep-water column from 1099 to 1219 m (Hazen et al., 2010). However, bacteria involved in organic pollutant degradation in deep-sea environments are largely unknown, especially in the open sea (Deming, 1998). In deep-sea water columns of the open Mediterranean Sea, various types of PAHs have been found sinking with suspended particles (Bouloubassi et al., 2006). The source and fate of PAHs in deep-sea environments are poorly understood. One possible source is hydrothermal activity. A variety of organic compounds, including PAHs and long-chain hydrocarbons, were detected in the hydrothermal fluids (McCollom and Seewald, 2007). These hydrocarbons may serve as organic carbon sources that are otherwise scarce in the deep-sea environments but needed for heterotrophic bacterial growth.

**FIGURE 5:**
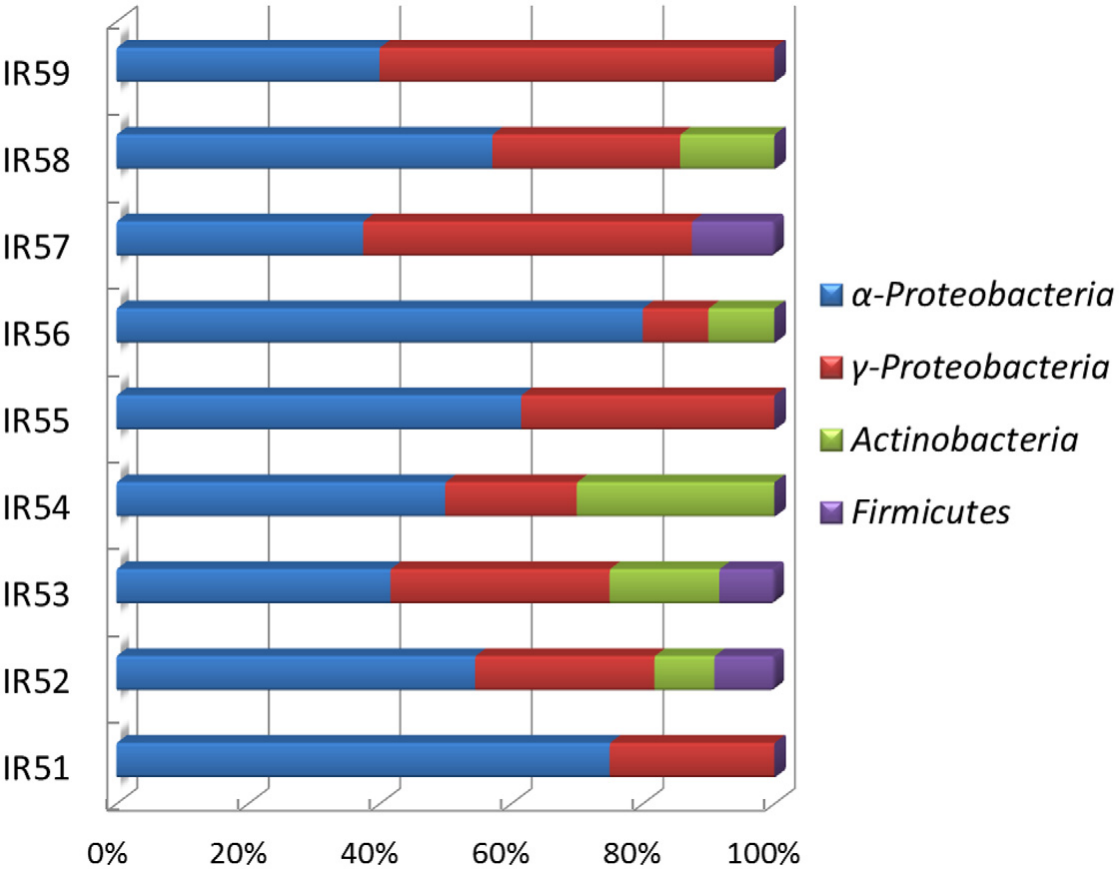
**Phylogenetic distributions of bacteria detected by DGGE in nine PAH-degrading consortia**.

Along the SWIR, hydrothermal vent fields were recently found (German et al., 1998; Tao et al., 2012). The microbial diversity of this region remains poorly characterized, and the bacterial diversity with regards to PAH degradation is unclear (Chen and Shao, 2009; Cao et al., 2014; Li et al., 2014). In this report, a variety of PAH-degrading bacteria were found in the deep water column on the Ridge at depths ∼4766 m. Among the discovered bacterial phylotypes, *Proteobacteria* dominated all the consortia, which was constituted of mainly *Alphaproteobacteria* and *Gammaproteobacteria* (**Figures 3** and **5**). Interestingly, the predominant PAH degraders obtained from the deep-sea water were significantly different from those in marine sediments. *Cycloclasticus*, thought to be the key PAH-degrading bacterium in marine environments, was not as important as previously reported (Dyksterhouse et al., 1995; Wang et al., 1996, 2008; Kasai et al., 2002; Yakimov et al., 2005; McKew et al., 2007; Teira et al., 2007; Cui et al., 2008). Instead, *Novosphingobium* seemed to be most important genus in this report. Bacteria of this genus were also previously detected as PAH degraders in the deep-sea hydrothermal environment of the Lau Basin of the West Pacific (Dong et al., 2011). They are closely related to those detected in this report, belonging to *N. indicum*, which we isolated here from sample CTD-5 (Yuan et al., 2008, 2009). This bacterium can degrade many types of aromatic hydrocarbons including biphenyl, naphthalene, acenaphthene, 2-methylnaphthalene, dibenzofuran, dibenzothiophene, 2,6-dimethylnaphthalene, 4- methyldibenzothiophene, phenanthrene, anthracene, chrysene, and fluoranthene (Yuan et al., 2008). However, contrary to expectations, only phenanthrene could be degraded alone, while the three other PAHs (anthracene, fluoranthene, and pyrene) could not be individually utilized by any of the consortia, as evidenced by the lack of clear bacterial growth within 1 month. We propose that phenanthrene metabolism induced the bacterial cells to utilize the three other PAHs more rapidly. This possibility is supported by a previous report, which found that pyrene degradation relies on the presence of phenanthrene in the case of the isolate *Cycloclasticus* sp. N3 (Geiselbrecht et al., 1998).

By comparing the bacterial structure of these consortia, a trend in bacterial distribution from 20 to 820 m above the bottom can be observed. Consortia from neighboring layers were similar in bacterial structure. For example, in phenanthrene-enriched consortia, the community structures of the upper two layers were similar to each other but different from those of the other layers (Supplementary Figure S3). Some bacteria occurred only in specific consortia. For instance, the bacterium closely related to *N. aromaticivorans* DSM 12444 (100%) occurred from 470 to 620 m above the bottom as bands P5-4, P6-5 and P7-5, but was not found in other layers (**Table 1**). The bacterium similar to *Azospirillum* sp. 5C (95.21%) occurred below 470 m from the bottom as bands 1–8, 3–10, 4–10, and 5–14, as did the bacterium similar to *Rhodovulum* sp. AR2002 (95.21%), which frequently occurred as bands 1–7, 2–11, 3–12, 5–13, P1–8 and P3–11 (**Table 1**). However, the strain that displayed 96.75% similarity with *Rhodovulum* sp. AR2002 was detected in a wider range of 20–820 m above the bottom, presenting as bands 1–6, 2–10, 5–12, 9–7, 6–10, P6–10, and P9–6 (**Table 1**). Additionally, the bacterium similar to *N. hassiacum* W-51 (95.81%) only occurred in the first and second layers above the bottom as bands 2–4, P1–4, and P2–6, while the bacterium similar to *N. pentaromativorans* US6-1 (98.64%) only occurred in the bottom layer as a key member of the PAH mixture consortia. However, the bacterium similar to *A. dieselolei* B-5 (99.47%) frequently occurred in all layers, and the bacterium similar to *Bartonella* sp. AD273 (98.79%) occurred in many consortia regardless of layer. Among these bacteria, *Alcanivorax*, and *Novosphingobium* have been reported in deep-sea sediments involved in PAH degradation (Cui et al., 2008; Wang et al., 2008; Cui and Shao, 2009). Recently, we found that PAH-degrading bacteria, including *Cycloclasticus*, *Pseudomonas*, *Pseudoalteromonas, Halomonas*, *Marinomonas*, and *Dietzia*, were widespread in the deep-sea sediments of the Arctic Ocean (Dong et al., 2015). In addition, *Alcanivorax* frequently occurred in the Arctic PAH-degrading consortia, while bacteria *Novosphingobium* did not.

Among the bacteria detected in this report, the bacterium similar to *A. dieselolei* B-5 (99.47%) was a key member of all consortia. The predominance of *A. dieselolei* was further confirmed by 16S rRNA gene library construction of two communities, IR58 and IR59 (data not shown). From the library, we obtained a nearly full length 16S rRNA, which showed 99.8% (1502/1505) similarity with the type strain *A. dieselolei* B-5. Moreover, *Alcanivorax* bacteria appeared in both the PAH mixture and the phenanthrene-degrading consortia. It has been demonstrated that *A. dieselolei* is an obligate alkane-degrading bacterium (Liu and Shao, 2005; Wang et al., 2010). However, bacteria of this genus have never been shown to be PAH degraders. Iwabuchi et al. (2002) suggested that *Alcanivorax* may enhance PAH degradation by utilizing the alkyl side-chains on methylated PAHs within crude oil (Iwabuchi et al., 2002). However, in this study, such alkyl side-chain-substituted PAHs were not used. Why *Alcanivorax* coexist with other bacteria in these communities remains an intriguing question.

Some bacteria grew better with the PAH mixture, while others preferred phenanthrene alone (**Table 1**). For example, the bacterium similar to *Zymomonas mobilis* (97.00%; Band2-6), the bacterium similar to *Halomonas aquamarina* DSM 30161 (100; Band9-6), the bacterium similar to *Microbacterium schleiferi* DSM 20489(100%; Band4–9), the bacterium similar to *Azospirillum* sp. 5C (95.21%), and the bacterium similar to *N. pentaromativorans* US6-1 (98.64%; Band1-3) occurred as major bands only in the PAH mixture. However, some were found only in the phenanthrene treatments, such as the bacterium similar to *Erythrobacter* sp.G265 (99.26%; Band P3–3), the bacterium similar to *Novosphingobium* sp. MG36 (100%; Band P6–6) and the bacterium similar to *Sphingomonas* sp. SAFR-042 (100%; Band P3-6) (**Table 1**). Recently, some of these bacteria have been reported to be PAH degraders in marine environments. For example, an isolate of *Microbacterium* from mangrove sediments (Wongwongsee et al., 2013) and *Erythrobacter* strain N3, isolated from south Atlantic deep-sea sediment (unpublished data from our group), can utilize phenanthrene to grow. A bacterium of *Sphingomonas* has also been reported in both soils and marine sediment involved PAH degradation (Nadalig et al., 2002; Uyttebroek et al., 2007). However, bacteria of *Zymomonas* and *Azospirillum* have not been confirmed yet in PAH degradation.

Conversely, some bacteria in this study occurred as major bands in both types of enrichment, including the bacteria similar to *N. aromaticivorans* (98.79, 100%), *Novosphingobium* sp. MG36 (98.77%), *Alcanivorax* sp. EPR 7 and *Alcanivorax dieselolei* B-5 (99.47%), and *Arthrobacter* sp. ADG1 (91.27%). Others occurred in both treatments but only appeared as a major band in the PAH mixture. These included the bacteria similar to *Arthrobacter* sp. 255-8a (96.46%), *Thalassospira lucentensis* (99.39%), *Novosphingobium hassiacum* W-51 (95.81%), *Rhodovulum* sp. AR2002 (95.21 and 96.75%), *S. oneidensis* (91.09%), and *C. spirillensus* (100%). Bacteria of different *Thalassospira* species have been previously detected, and then later confirmed, in PAH-degrading consortia (Liu et al., 2007; Kodama et al., 2008; Zhao et al., 2010). Many of these bacteria have not been successfully isolated on agar plates, such as *Cycloclasticus spirillensus*, and potentially novel species such as *Arthrobacter, Azospirillum*, and the *Rhizobiales* and *Rhodovulum* with 16S rRNA sequences 90–97% similar to related strains. *Azospirillum*, *Porphyrobacter*, *Shewanella*, and *Bartonella* also fall into this category. More efforts toward pure culture cultivation are needed to confirm their role in PAH degradation.

Among the PAHs used, phenanthrene is probably the easiest to degrade, as the resulted PAH-degrading consortia failed to show obvious bacterial growth when using pyrene, anthracene, or fluoranthene as the sole carbon source. However, when used in form a mixture, all of them can de degraded, suggesting a cometabolism between phenanthrene and other PAHs. This phenomenon has been found in other reports (Geiselbrecht et al., 1998; Kanaly and Harayama, 2000). In the case of pure cultures, 34 of the isolates have been confirmed to grow with the PAH mixture. By now, their degradation ability on each kind of PAHs remains undetermined except one novel species of *N. indicum* mentioned above (Yuan et al., 2008, 2009).

In respect of temperatures and hydrostatic pressures *in situ* that might select bacteria different to the above described, obligate psychrophilic and barophilic microbes involved in PAH degradation probably exist in the deep sea water column. Bacterial isolates in this report ever tested showed temperature growth ranges from 10 to 41°C mostly, with optimal growth temperatures of 20 to 25°C, such as *Alcanivorax marinus, N. indicum, Oceanibaculum indicum*, and *Stappia indica* et al., which are the novel species isolated from the deep sea water of SWIR (Lai et al., 2009a,b, 2010, 2011a,b, 2013; Yuan et al., 2009). They did not show an obvious growth under 4°C within 1 week, which is higher than the temperature (about 1°C) *in situ*. Probably, they actually grow slightly under temperature at 4°C and below, or need long time incubation. Anyhow, we believe that microbes *in situ* would possess some taxa that are psychrophilic and barophilic, but remain uncultivated by now.

In summary, this report describes the discovery of diverse PAHs-degrading bacteria in the deep-sea water column of the SWIR at a depth of 4766 m. Bacteria affiliated with *Alcanivorax, Novosphingobium*, and *Rhodovulum* frequently occurred in different PAH-degrading consortia, and 34 isolates were confirmed to grow with our PAH mixture. These included novel PAH-degrading bacteria belonging to *Alterierythrobacter, Citricella, Erythrobacter, Idiomarina, Lutibacterium, Maricaulis, Marinobacter, Martelella, Pseudidiomarina, Rhodobacter, Roseovarius, Salipiger, Sphingopyxis*, and *Stappia*. These bacteria are thought to play a role in PAH degradation in deep-sea water. More efforts are needed to examine the diversity of extremophiles *in situ* of deep sea that involved in PAH degradation.

## Author Contributions

ZS: conceived and designed the project, oceanic sampling and on board enrichment. JY, QL: bacterial isolation, identification, DGGE and GS-MS analysis. FS: bacteria management. ZS, YJ, and TZ: writing the paper.

## Conflict of Interest Statement

The authors declare that the research was conducted in the absence of any commercial or financial relationships that could be construed as a potential conflict of interest.

## Supplementary material

FIGURE S1 **Sampling site IR-CTD5 (The map based on NOAA topography).** The solid red circles are hydrothermal vent fields previously detected. IR-CTD5 was in the B-B’ section, where plume 2 was found by anomalies (German et al., 1998). Edmond and Kairei are confirmed vent sites.

FIGURE S2 **Community structures of the PAH-degrading consortia growing with 4-PAH mixture in 20 days.** Derived from water samples of IR5-2 to IR5-9 after PAH enrichment, with water depths of 4696, 4546, 4396, 4296, 4196, 4146, 4096 and 3946 m, and 40–820 m above the sea floor.

FIGURE S3 **Community structures of the PAH-degrading consortia growing with phenanthrene in 20 days.** Treatments were in parallel with those in Figure S2, except using phenanthrene as a sole carbon source.
