# Supplementary figures and images for: The diversity of PAH-degrading bacteria in a deep-sea water column above the Southwest Indian Ridge
Source: Front Microbiol. 2015 Aug 25;6:853. doi: 10.3389/fmicb.2015.00853 (PMC4548250; doi:10.3389/fmicb.2015.00853)

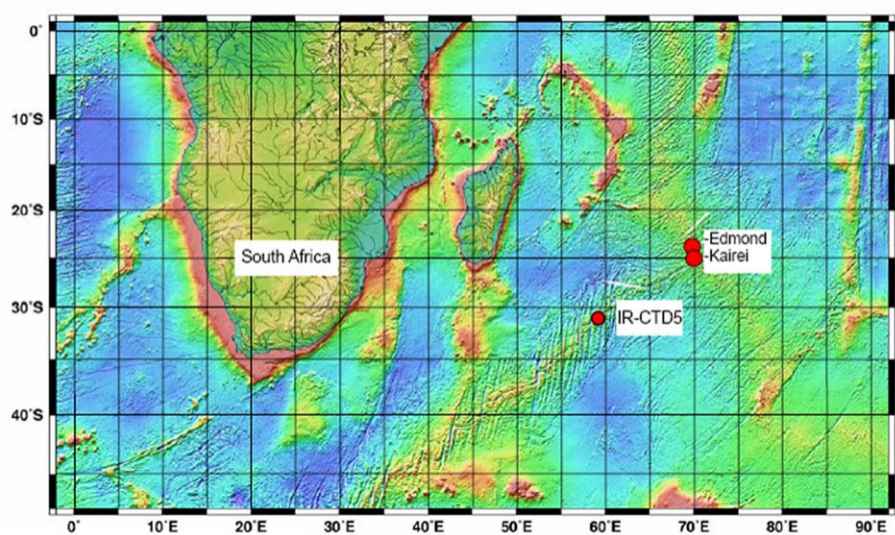

Supplement: FIGURE S1 — Sampling site IR-CTD5 (The map based on NOAA topography). The solid red circles are hydrothermal vent fields previously detected. IR-CTD5 was in the B-B’ section, where plume 2 was found by anomalies (German et al., 1998). Edmond and Kairei are confirmed vent sites. [file Image_1.PDF]

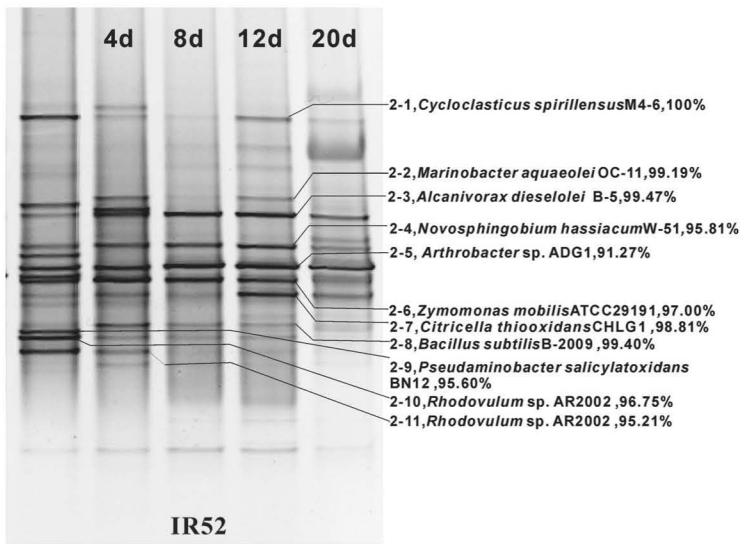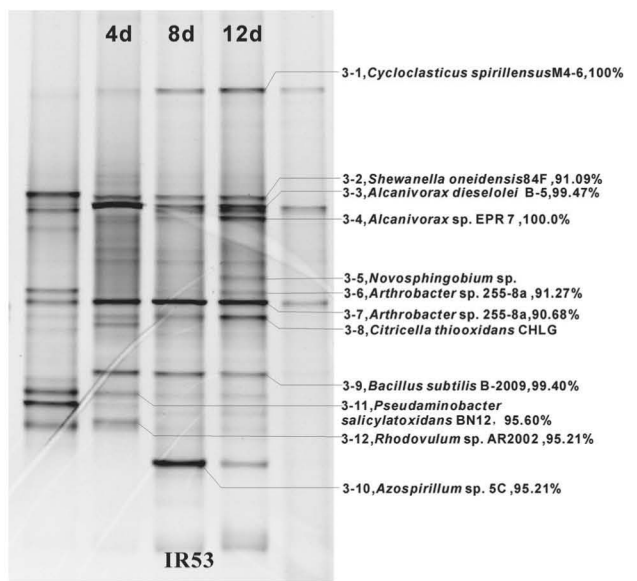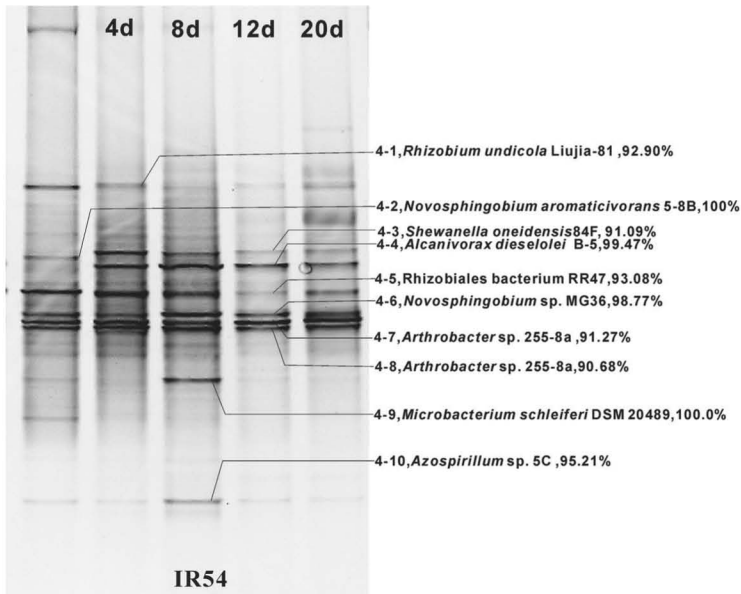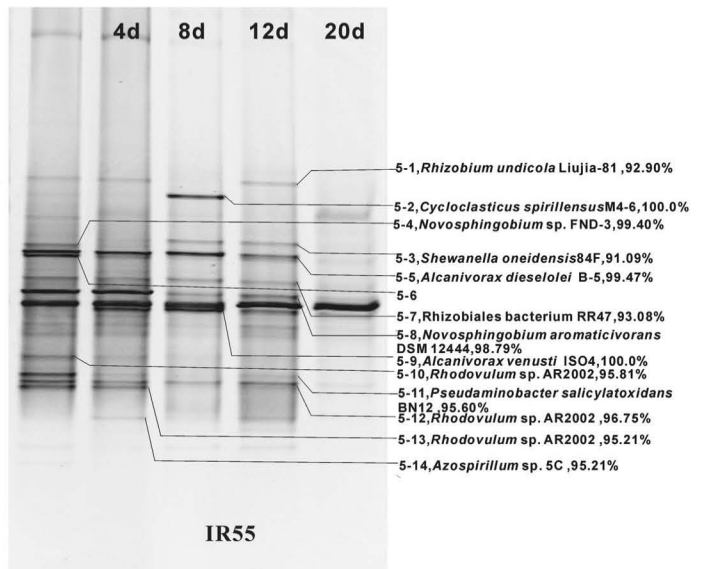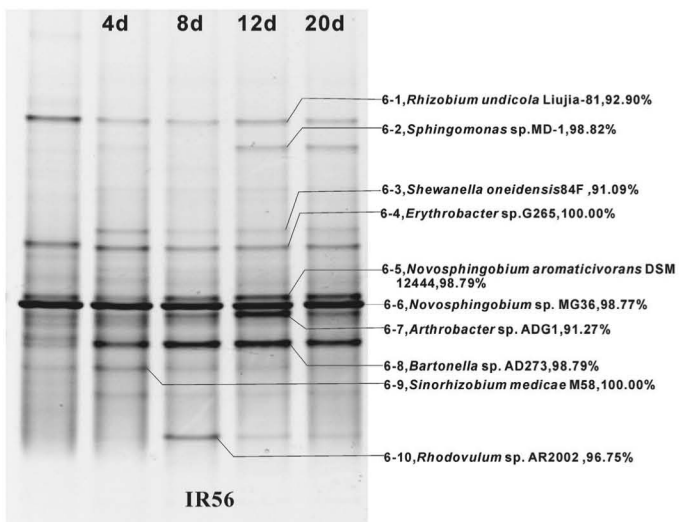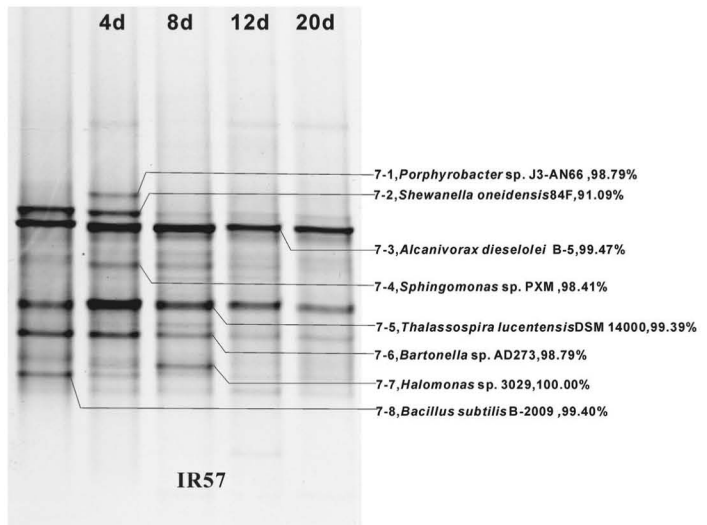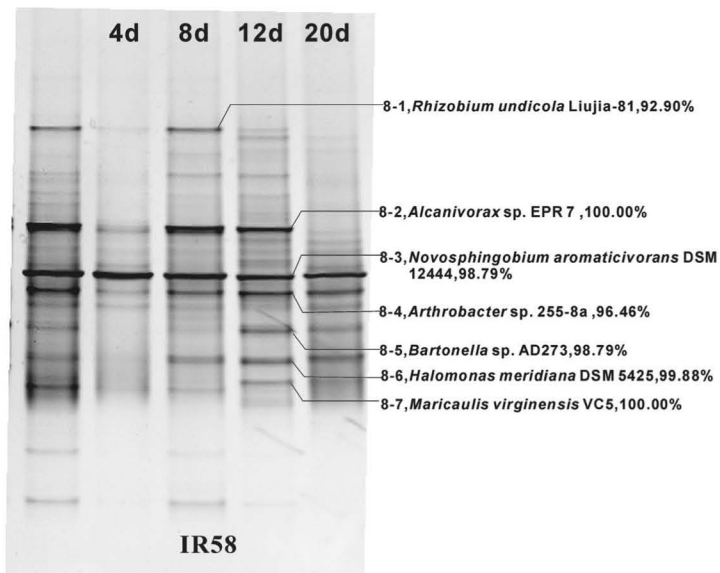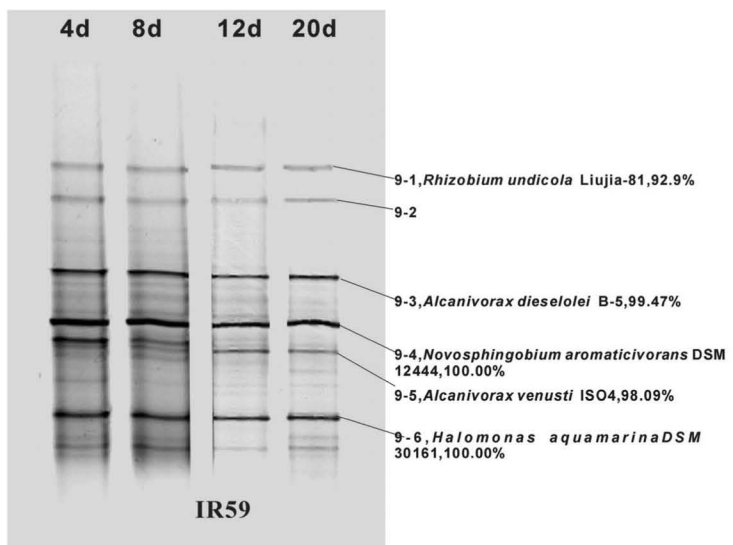

Supplement: FIGURE S2 — Community structures of the PAH-degrading consortia growing with 4-PAH mixture in 20 days. Derived from water samples of IR5-2 to IR5-9 after PAH enrichment, with water depths of 4696, 4546, 4396, 4296, 4196, 4146, 4096 and 3946 m, and 40–820 m above the sea floor. [file Image_2.PDF]

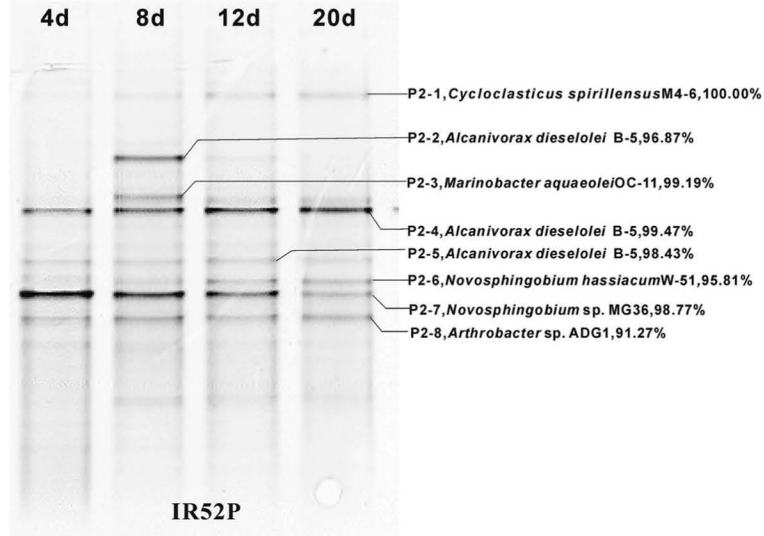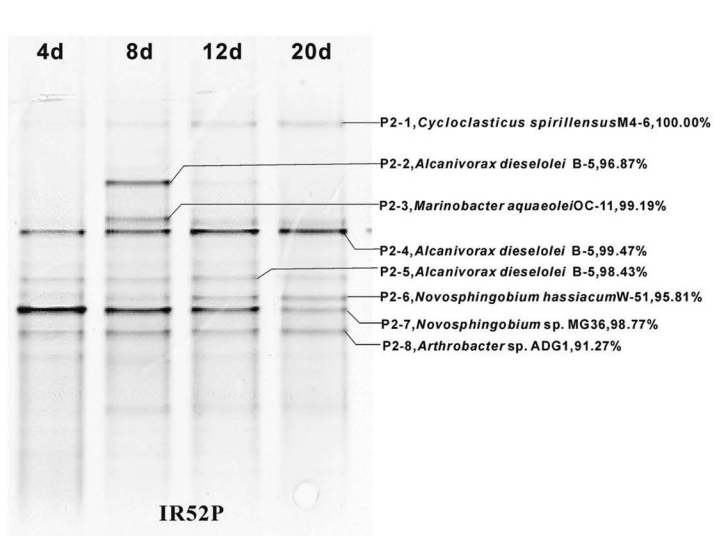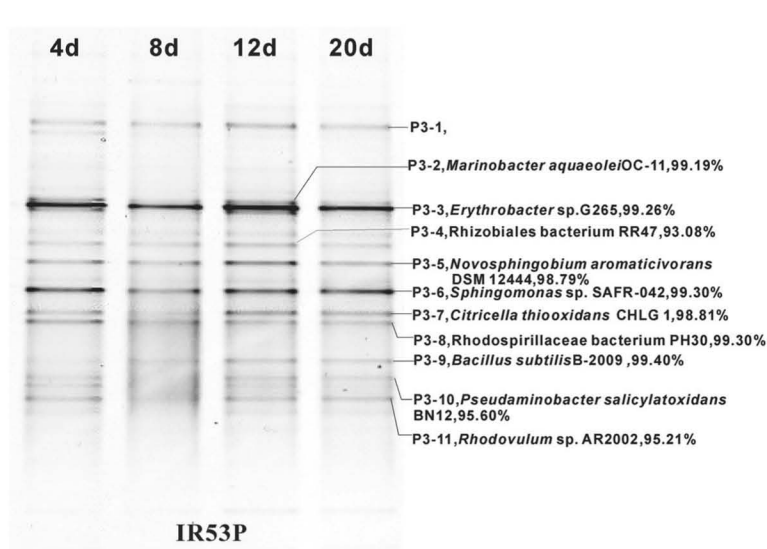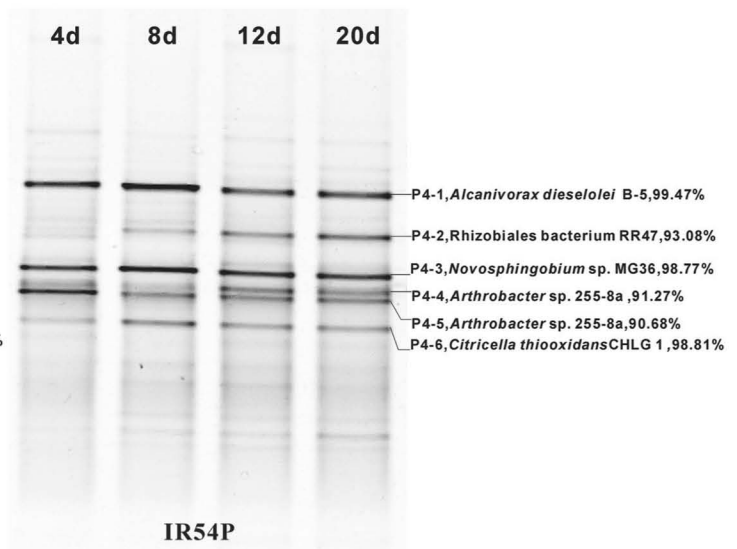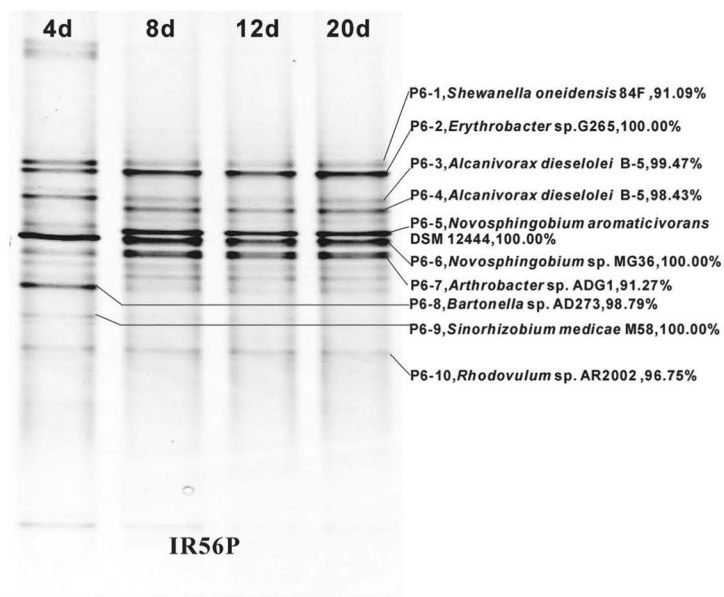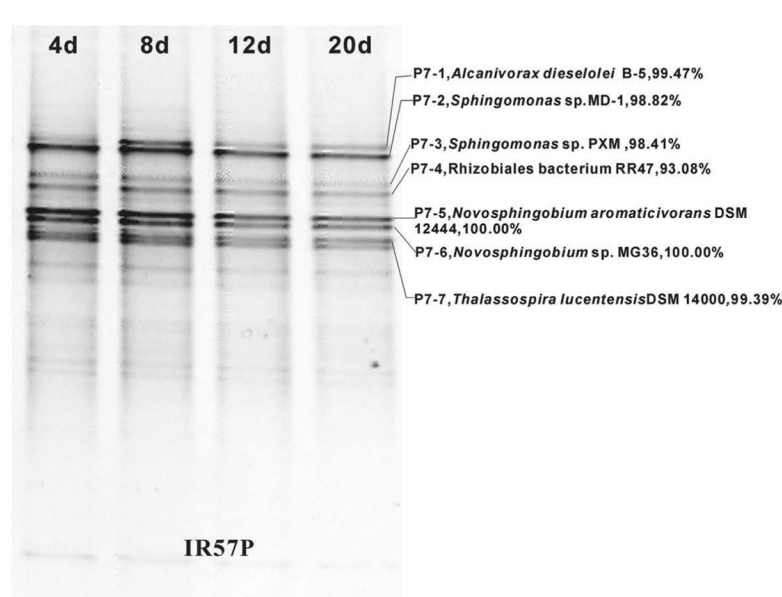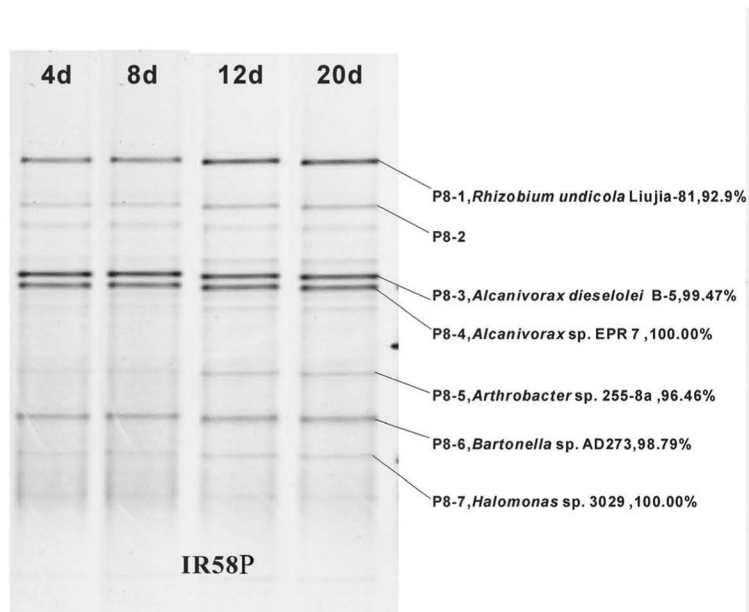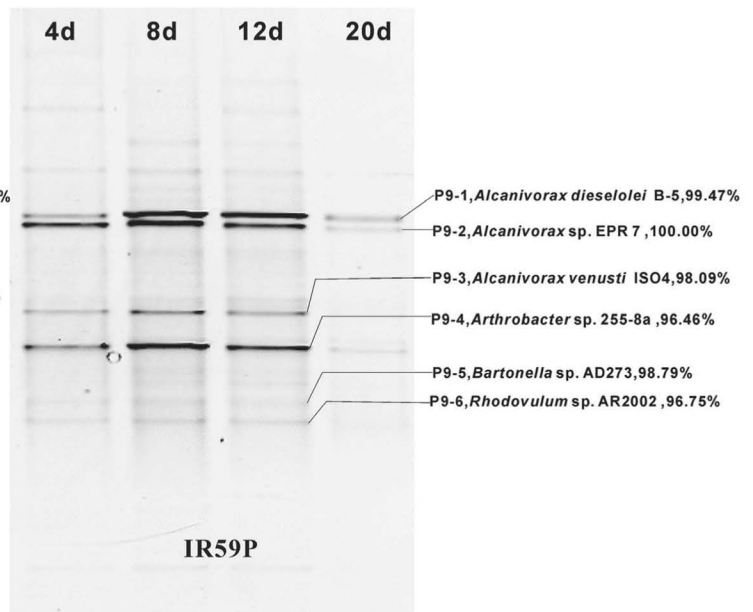

Supplement: FIGURE S3 — Community structures of the PAH-degrading consortia growing with phenanthrene in 20 days. Treatments were in parallel with those in Figure S2, except using phenanthrene as a sole carbon source. [file Image_3.PDF]
